# Impact of medication review via tele-expertise on unplanned hospitalizations at 3 months of nursing homes patients (TEM-EHPAD): study protocol for a randomized controlled trial

**DOI:** 10.1186/s12877-020-01546-3

**Published:** 2020-04-20

**Authors:** F. Correard, M. Montaleytang, M. Costa, M. Astolfi, K. Baumstarck, S. Loubière, K. Amichi, P. Auquier, P. Verger, P. Villani, S. Honore, A. Daumas

**Affiliations:** 1 Service pharmacie, hôpital de la Timone, Assistance Publique des Hôpitaux de Marseille (AP-HM), Marseille, France; 2 Aix-Marseille Univ, Marseille, France; 3 ORS PACA, Southeastern Health Regional Observatory, Marseille, France; 4 EA3279, Self-perceived Health Assessment Research Unit, Marseille, France; 5 Direction de la Recherche Clinique et de l’Innovation (DRCI), AP-HM, Marseille, France; 6 IRD, AP-HM, SSA, VITROME, Marseille, France; 7 IHU-Méditerranée Infection, Marseille, France; 8 Internal Medicine, Geriatrics and Therapeutics department, AP-HM, Marseille, France

**Keywords:** Drug, Elderly, Iatrogenic, Inappropriate medication, Telemedicine, Tele-expertise

## Abstract

**Background:**

Inappropriate drug prescribing causes preventable drug-related adverse events that result in increased morbidity and mortality, additional costs and diminished quality of life. Numerous initiatives have been launched to improve the quality of drug prescribing and safeguard the security of drug administration processes in nursing homes. Against the backdrop of implementation of telemedicine services, the focus of the present work is to evaluate the impact of a telemedication review carried out by a hospital physician and pharmacist as part of the telemedicine offer.

**Methods:**

The present study is a randomized controlled clinical trial. A total of 364 patients will be randomized into two groups: (1) an experimental group (182 patients) benefiting from a telemedication review using tele-expertise and (2) a control group (182 patients) receiving standard care. The primary endpoint will be rate of all-cause unplanned hospital admissions occurring within 3 months of randomization. The secondary endpoints will be rate of unplanned admissions at 6 months, patient quality of life, incidence of behavioral disturbances, number of falls, number of residents prescribed at least one inappropriate medication, nursing staff satisfaction, proposed medication reviews and their acceptability rate, characteristics of patients whose general practitioners have taken account of tele-expertise, efficacy of tele-expertise as compared to standard prescription and acceptability and satisfaction surveys of participating caregivers.

**Discussion:**

In the literature, various studies have investigated the utility of structured medication review processes, but outcome measures are heterogeneous, and results vary widely. Medication review can detect medication-related problems in many patients, but evidence of clinical impact is scant. Incremental cost-effectiveness ratios will be used to compare the cost and effectiveness of the experimental strategy and that of standard care. Our approach, involving the combination of an acceptability survey and a mixed-method (qualitative and quantitative) satisfaction survey, is particularly innovative. The results of this randomized trial are expected to confirm that medication review using tele-expertise has potential as a worthwhile care management strategy for nursing home residents.

**Trial registration:**

Clinicaltrials.gov NCT03640845; registered August 21, 2018 (Clinicaltrials.gov NCT03640845).

## Contributions to the literature

- Our paper will determine the impact of telemedication review at high iatrogenic risk on unplanned hospital admission in addition to health-related quality of life, behavioral disturbances, frequency of falls, potentially inappropriate medications and convenience of drug administration for nursing staff
- We will provide insight into the impact of tele-expertise in terms of health economics with a cost effectiveness study.
- Ultimately, we will attempt to determine which organizational and psychosocial factors are likely to facilitate or on the contrary to hamper telemedication review development and acceptability

## Background

One of the effects of population aging is increasing the risk of drug-induced iatrogenic disease for whom appropriate prescription is not just a matter of healthcare quality and safety but also one of its economic efficiency [1]. The challenge for the prescriber when treating older multimorbid patients is to strike a balance between optimizing chronic disease control and minimizing the risks of polypharmacy. In the elderly population, potentially inappropriate medication use, drug–drug interaction or drug–disease interaction are associated with negative health outcomes and contribute significantly to 6–17% of hospital admissions in older adults [2–5]. In France, medication-induced iatrogenic disease is believed to account for over 10% of hospital admissions in patients over 65 years of age, 20% of admissions in patients over 75 years of age and 25% of admissions in those over the age of 85 [6]. Given that 30 to 60% of adverse drug events are thought to be foreseeable and avoidable, improving the quality and safety of drug prescription is of paramount importance for public health, particularly in elderly patients [7].

In nursing homes, residents accumulate a mean of 8 comorbidities (85% of residents present neuropsychiatric disorders and 75% cardiovascular disease), take a mean of 8 different medications per day and roughly one third of them suffer from swallowing disorders [8, 9]. According to data analysis from an experimental project to re-establish medication as part of care plans offered by nursing homes with no in-house pharmacy in the Aquitaine and Limousin regions of France in 2012, 25% of residents were subjected to excessive treatment and 70% were found to have been prescribed medication unnecessarily. It is also necessary to add the omission of potentially appropriate medication (i.e. no drug prescription despite indication). Other factors also promote medication-induced iatrogenic disease in elderly patients, such as pharmacodynamic and pharmacokinetic alterations as well as multiple prescribers. Systematic review shows that almost one half of nursing home residents are exposed to potentially inappropriate medications and suggests an increase prevalence over time [10]. Effective interventions are therefore required to optimize drug prescribing in nursing home facilities.

Several recommendations aimed at addressing these concerns have been issued on a national scale [11–13]. Numerous initiatives have also been launched to improve the quality of drug prescribing and the security of drug administration processes in nursing homes, and these initiatives are supported by various nationwide schemes. Systematic medication review is a simple and effective way of minimizing inappropriate drug prescription and optimizing how medication is prescribed to this population [14, 15]. Within the framework of this approach, it is essential to obtain an accurate insight into a patient’s treatment with regard to the comorbidities and potential geriatric syndromes involved, the patient’s wishes and any available pharmacological evaluation tools, such as STOPP/START.v2 criteria or the Laroche list. Medication review in older adults must also be conducted in accordance with healthcare objectives centered primarily on patients and their independence; this is particularly applicable to patients who are frail, suffering from multiple comorbidities and/or very elderly. The suitability of each medication should be discussed in terms of expected benefits and risk of adverse events while also taking into consideration patient preference and life expectancy. The main purpose of this type of review is to optimize drug prescription and ensure that more good is done than harm.

Nowadays telemedicine embodies a new way of structuring medical practice. It is a potential vehicle for better access to healthcare since it overcomes patient transport difficulties and mitigates the chances of a patient refusing healthcare (for geographical, economic or social reasons), thereby improving quality of care (in terms of avoidable hospital admission and reduced utilization of emergency services) and minimizing use of healthcare resources (such as healthcare consumption and transport costs) [16]. Tele-expertise (TE) allows a doctor, dubbed the “requesting” physician, to ask advice on the strength of specialist training or skills from a colleague, dubbed the “consulting” physician, based on details or elements from a patient’s medical records, without the patient’s physical presence. Until the end of 2020, TE in France will only apply to patients requiring help with access to healthcare on account of their state of health or geographical circumstances; residents from nursing homes fall into this category. Accounts of telemedicine found in the literature essentially focus on TE (recommendations on findings from follow-up examinations related to dermatological problems or chronic wounds for example) and telemonitoring for chronic disease such as heart failure [17–20]. To our knowledge, there are no telemedication review (TMR) in the literature. The purpose of the present study is to evaluate the impact of TMR regarding residents at high iatrogenic risk versus standard care on unplanned hospital admission rates at 3 and 6 months in addition to health-related quality of life, behavioral disturbances, frequency of falls, potentially inappropriate medications and convenience of drug administration for nursing staff. This study will also provide an assessment of the impact of TE in terms of health economics. Finally, a qualitative study conducted by the regional health observatory will contribute to a better understanding of the organizational and psychosocial factors associated with the success of this approach or conversely those that have hampered its acceptability and its development.

## Methods

### Study design

This prospective, multicenter, single-blind, randomized, parallel-group trial is conducted in accordance with the ethical principles of the Declaration of Helsinki and the principles of current Good Clinical Practices. The study protocol (Version 2, 29/10/2019) is designed in accordance with guidelines from the Consolidated Standard of Reporting Trials (CONSORT) statement.

### Recruitment and randomization

Recruitment will take place within consenting nursing homes contacted by the gerontology coordination groups of Marseille. Nursing home coordinating physicians will act as study investigators in charge of patient inclusion. Written consent to participate will be obtained at the Nursing home after the investigator has provided all necessary information about the study. Randomization will be performed at patient level, with eligible patients randomly assigned in a 1:1 ratio to the control group (non-intervention) or the experimental group (TMR) based on a computer-generated permuted-block randomization schedule with blocks of four. Randomization is to be stratified by an adverse drug event risk detection score in elderly patients, the Trivalle’s risk score (≤ 5 or > 5). Based on the IMEPAG study [21], this score is both easy and practical to use for detecting the risk of adverse drug events in elderly patients. Risk factors included in the score are: number of medications (if greater than or equal to 7), antipsychotic treatment and recent anticoagulant treatment (dating back less than 3 months). Each parameter is scored on a scale from 0 to 10, with 0–1 corresponding to an adverse drug event risk of 12%, 2–5 corresponding to a 32% risk and 6–10 to a 53% risk.

Computer-generated randomized lists will be drawn up before the beginning of the study under the responsibility of the local Clinical Research Unit. Methodological support will be provided by the Clinical Research Unit. Study investigators will not be blinded to group allocation. The recruitment period will span 20 months, and patient follow-up will last 6 months (Table 1).

**Table 1:** Timeline of the study

Timepoint	Study Period			
	Enrolment	Inital assessment	Follow-up at 3 months	Follow-up at 6 months
**Enrolment**				
Eligibility screen	X			
Information to patients/careviger	X			
Collection of consent	X			
Date of the visits	X	X	X	X
**Assessments**				
*Primary outcome:*				
Unplanned Hospitalizations			X	
*Secondary outcomes:*				
Unplanned Hospitalizations				X
Behavioral disorders (NPI)		X	X	X
Quality of life (EQ-5D-3 L)		X	X	X
Number of falls		X	X	X
Number of patients with at least 1 potentially inappropriate prescription		X	X	X
Nursing staff satisfaction		X	X	
Characteristics of patients whose GP have taken account of TMR recommendations			X	X
GP’ adherence rate to therapeutic recommendations			X	X
Cost-effectiveness ratio of TMR				X
Factors facilitating or hampering GPs’ adherence rate		X	X	X
**Characteristics**				
Sociodemographic data (age, gender)	X	X		
MMSE	X			
Comorbidities		X	X	X
Pharmaceutical therapeutics		X	X	X
Blood test		X	X	X
Trivalle’s score	X			

### Inclusion criteria

Predefined inclusion criteria are that participants must: be at least 65 years old; live in a nursing home; have a Trivalle’s score of between 2 and 10; and be affiliated to the French public welfare system.

The patient or informal caregiver or legal representative will sign the informed consent form prior to randomization.

### Exclusion criteria

Patients who exhibit any of the following criteria will be excluded: life expectancy < 3 months; involvement in another research project; medication review conducted by a pharmacist in the last 6 months; mini mental state examination (MMSE) score < 18 [22].

Refusal to participate on the part of general practitioner (GP) constitutes a secondary exclusion criterion in this study.

### Ethical and legal considerations

The protocol has been approved by the regional ethics committee (*Comité de protection des personnes Sud Est II*) on 21 November 2018. Data processing has been approved by the national data protection commission.

### Sample size

**Fig. 1:**
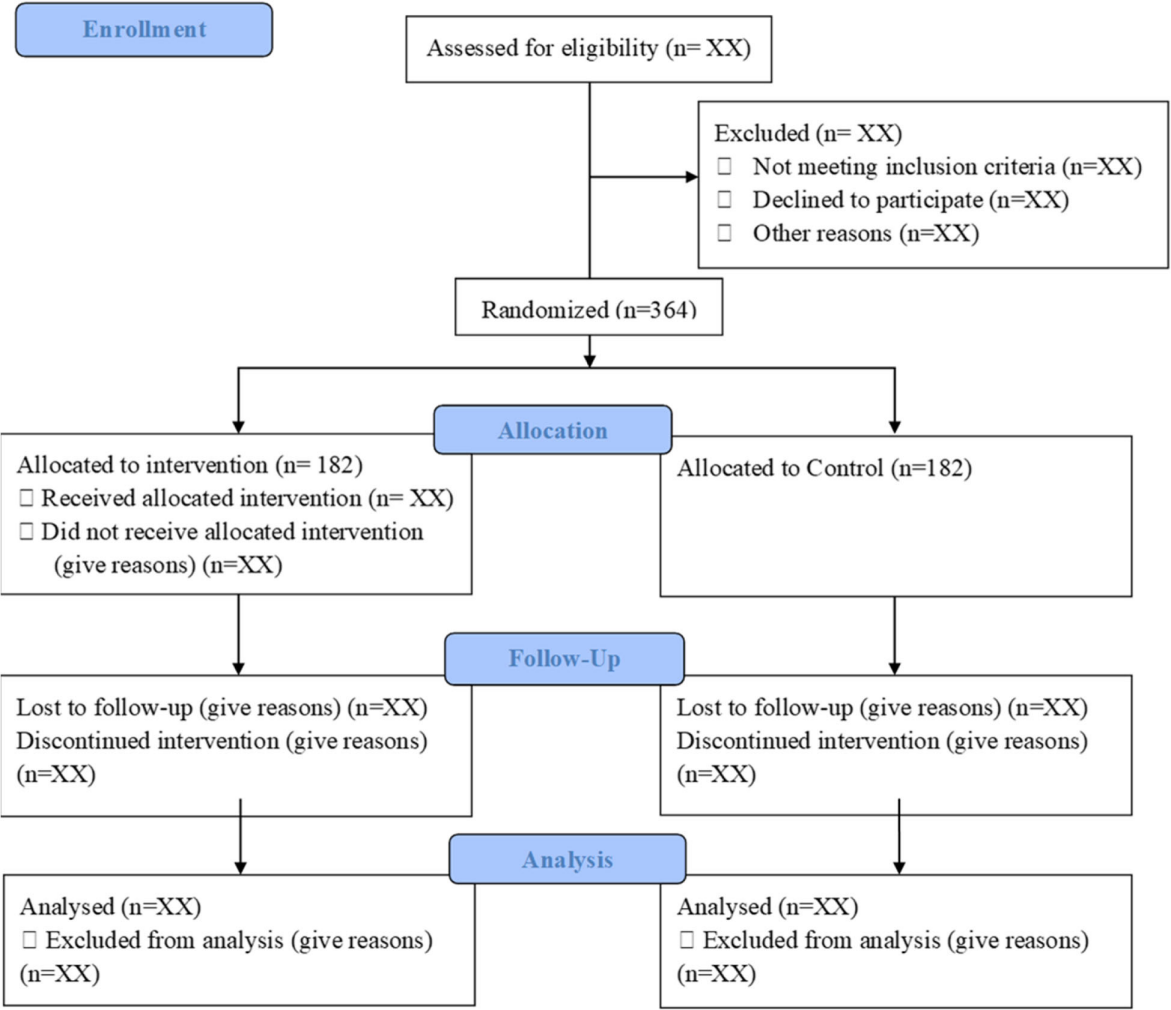
Flow diagram of the study protocol

On the basis of a unplanned hospital admission rate of 25% over a 3-month period [23] and taking into consideration the results of a study evaluating the impact at 8 weeks of medication review on admission to nursing home [24], we estimate a unplanned hospital admission rate of 10% in the experimental group. The initial sample size of 292 patients provided 90% power to detect this difference at a two tailed significance level of 0.05. Assuming possibly 25% of patients lost to follow-up, a total of 364 patients is required (182 per group) (Fig. 1).

### Groups

#### Experimental group

Patients randomized into experimental group will benefit from TMR undertaken by a hospital-based team composed of a clinical pharmacist and a geriatrically-trained internal medicine specialist. For the purposes of issuing quality validation and relevant guidelines, TMR requires:

- medication reconciliation to have an exhaustive list of all medications taken by the patient (including prescription and non-prescription medication);
- inclusion of the most recent results from laboratory tests (blood sodium, potassium and creatinine levels) and clinical data (blood pressure, weight);
- knowledge of patient history, active comorbidity and geriatric syndromes.

TMR means undertaking multidisciplinary medication reviews and taking into consideration all available sociodemographic, clinical, biological and pharmaceutical data pertaining to patients to optimize their therapeutic management.

It should be noted that an exhaustive list of patient medications will be acquired through medication reconciliation in accordance with the guidelines of French National Authority for Health [25].

Each drug prescription will be assessed in light of patient medical conditions, level of dependence and quality of life (amount, dosage, route of administration, contra-indications, drug interaction, and precautions for use). Medication databases will be consulted, following which adjustments to prescriptions can be made using the explicit criteria from STOPP/START.v2 criteria coupled with an implicit approach (the Medication Appropriateness Index) and taking into consideration existing therapeutic guidelines. A written report summarizing any proposed therapeutic adjustments will be produced and officialized as a personalized pharmaceutical plan (PPP) signed by both physician and pharmacist. The conclusions resulting from TMR will be transmitted to healthcare professionals within a maximum of 5 days after patients have been randomized to the experimental group. Should a therapeutic error with repercussions for patient prognosis be detected, the coordinating physician and patient’s GP will be informed immediately by phone. The GP can accept or reject the medication adjustments proposed by TMR. Data relevant to the study will be collected at the inclusion, 3 and 6 months.

#### Control group: standard care management

Control group patients will receive standard care from the medical and paramedical team of the nursing home where they reside. No medication reconciliation or medication review of their prescribed drugs will be conducted. Data relevant to the study will be collected at the inclusion, 3 and 6 months.

### Study aims

The objective of this study is to reduce unplanned hospital admission using TMR and determine which organizational and medico-psychosocial factors facilitating or hampering TMR development.

#### Primary endpoint

The primary endpoint is the rate of all-cause unplanned hospital admission occurring within 3 months of randomization. All-cause hospital admission is defined as a unplanned hospital stay in a medical or surgery department or intensive care unit, irrespective of reason for admission or duration of stay.

#### Secondary endpoints

Assessment of unplanned hospital admission at 6 months post-randomization will be complemented by the following endpoints:

- Number of unplanned hospital admissions at 6 months post-randomization.
- Quality of life (QoL) of nursing home residents will be evaluated by the validated french version of EuroQol five-dimension three-level (EQ-5D-3 L) generic quality-of-life questionnaire [26, 27]. The questionnaire consists of two parts: the EQ-5D descriptive system and the EQ visual analogue scale (EQ VAS). The EQ-5D-3 L descriptive system comprises five dimensions as follows: mobility, self-care, usual activities, pain/discomfort and anxiety/depression. Each dimension has 3 levels: no problems, some problems, and extreme problems. The patient is asked to indicate his/her health state by ticking the box next to the most appropriate statement for each of the five dimensions. Each response generates a 1-digit number that represents the level selected for that dimension. The EQ VAS records self-rated patient health on a vertical visual analogue scale where the endpoints are labelled ‘best imaginable health state’ and ‘worst imaginable health state’. The EQ-5D-3 L questionnaire will be administered at the inclusion, at 3- and 6-month follow-up.
- Incidence of behavioral disturbances will be assessed by the validated french version of Neuropsychiatric Inventory (NPI) questionnaire at the inclusion, at 3- and 6-month follow-up [28]. NPI scoring is based on interviews with main caregivers. The NPI assesses the frequency (4-point scale) and severity (3-point scale) of 10 neuropsychiatric disturbances (delusions, hallucinations, agitation, dysphoria, anxiety, euphoria, apathy, disinhibition, irritability, and aberrant motor behavior), and a score from 0 to 12 is obtained for each scale by multiplying frequency by severity. The total NPI score is the sum of the subscale scores.
- The number of falls at 3- and 6-months post-randomization.
- The proportion of residents who will have been subjected to at least 1 potentially inappropriate prescription at the inclusion, 3 and 6 months. STOPP and START tool will be used for explicit criteria (French version 2). The implicit approach includes all available medical data, potential self-medication, and questions from the Medication Appropriateness Index [29].
- Nursing staff satisfaction with methods of medication administration to residents, number of oral medications dispensed, choice of galenic formulation, time spent on drug administration, and treatment dispensed to a patient consistent with that patient’s state of health. Satisfaction will be evaluated using 5-point Likert scales at the inclusion and at 3 months.
- Description of therapeutic recommendations which will have been proposed and GP’ adherence rate of its at 3- and 6-months.
- Characteristics of patients whose GP have taken account of TMR recommendations.
- Cost-effectiveness ratio of TMR versus standard care at 6-month follow-up (see details in Health Economic Analysis section below).
- The present study will also attempt to determine which organizational and psychosocial factors are likely to facilitate or on the contrary to hamper TMR development and acceptability (see details in Evaluation of Acceptability/Satisfaction section below).

### Study procedures

The evaluation will be performed at three different time points: baseline, 3 and 6 months after the randomization (Table 1). Data collection will be conducted by a clinical research assistant and reported in the Case Report Form.

The source of this data will be resident medical records and visits to residents and their care providers to complete the questionnaires. Participant’s file and electronic data are store security of the study site. Patient data are only identifiable with the unique participant’s hospital registration number. This variable will be deleted once the data is completed making the dataset anonymous. This process is in accordance to MR-001 methodology.

All adverse events will be declared to the promotor within 24 h. Causal relationship between adverse events and TMR will be analyzed.

### Data management and analysis

Data will be monitored by a clinical research monitor. Inconsistencies will be reported to the study investigators in order to decide whether the data should be corrected or considered as missing data. Statistical analysis will be performed after checking the database. After freezing, the consolidated data will be processed by statistician. The modified intention-to-treat population (including all randomized subjects evaluated at least at baseline; patients having withdrawn consent will be excluded from the final analysis) will be used in the primary analysis. No interim analysis is planned. Baseline parameters will be presented by group (control and experimental). No comparisons will be made in accordance with CONSORT guidelines.

#### Primary endpoint

All-cause unplanned hospital admission rates (see Primary Endpoint above) will be compared between the 2 groups (control vs experimental) using Chi-square or Fisher’s exact tests (as primary analysis). The results will be summarized as relative risks and its 95 95% CI.

As secondary analysis: i) survival estimates will be calculated in line with the Kaplan–Meier method and compared using a log-rank test; ii) adjusted analysis will be applied to potential confounding factors (difference observed in baseline characteristics) using logistic regression. A potential center effect will be assessed by mixed-effects modeling using generalized linear mixed model methodology (SAS software, 9.4 version, GLIMMIX procedure, center as a random effect). The result will be summarized in terms of odd ratios and associated 95% CI; iii) heterogeneity of the effect among pre-specified subgroups will be tested using the hazard ratio test and 95% CI, provided per subgroup.

#### Secondary endpoints

Comparisons between the 2 groups were performed for the secondary endpoints using Chi-square or Fisher’s exact tests for proportions and Student t or Mann-Whitney tests for continuous variables.

### Health economic analysis

Incremental cost-effectiveness ratios (ICER) will be used to compare the cost effectiveness of the experimental strategy with that of standard care. The ICER is the ratio of the difference in costs between groups to the difference in effectiveness. As recommended by the French National Authority for Health [30], incremental effectiveness will be measured in terms of quality-adjusted life years (QALYs), which is particularly relevant in this instance since TE associated with a reduction in unplanned hospital admission rates would have immediate impacts on patient QoL. These QALYs are determined by multiplying life years by a utility value associated with the health states experienced (corresponding to patient quality of life) during the period under consideration. Preference-based utility scores will be calculated using the EQ-5D-3 L questionnaire [26, 27].

The cost perspective taken in our economic analysis is that of the healthcare payer. The time horizon starts at the first baseline evaluation and ends at 6-month follow-up. This horizon is pertinent for recording immediate and relevant healthcare utilization and costs associated with the innovative strategy and represents a period during which all effectiveness and cost data will be accurately collected. The healthcare resources included will be those that are likely to differ between the experimental and control groups and will consist of: the TE intervention (including all categories of staff involved in data analysis and PPP writing, equipment required, time taken by coordinator/physician to collect and transfer data) and the healthcare costs generated by follow-up (including pharmaceutical medications and associated treatments, unplanned consultations and examinations, emergency department visits, inpatient stays differentiating between planned and unplanned hospital admissions, and transport costs). All resources will be collected from patient medical records and where necessary from referred hospital Departments of Medical Information (DIM) to identify exhaustive use of inpatient resources. Unit costs will be estimated using data from the French National Hospital Database and the national tariff. Medication costs will be obtained from the French Register of Pharmaceutical Specialties, an online database providing information on healthcare products. All resources will be valued in 2020/2021 euros, and the 6-month trial period means that no application of discount is required.

Descriptive statistics will be used to analyze effectiveness and cost variables, being both univariate and multivariate analysis (using non-parametric tests and bootstrap procedures respectively). Reweighted survival estimators and repeated measures of health-related QoL will be used in case of censored data. Probabilistic sensitivity analyses, using the non-parametric bootstrap method, will be carried out to generate mean expected ICERs and to determine whether uncertainty or variation in the data used affect the ICERs [31]. In addition, cost-effectiveness acceptability curves will be constructed to represent decision uncertainty surrounding cost-effectiveness estimates [32].

### Evaluation of acceptability/satisfaction

An acceptability/satisfaction survey will be conducted using a multiple phase mixed-method design [33]. Several data collection phases will occur sequentially as follows:

Firstly, a pre-intervention acceptability survey will be carried out to determine care provider acceptance and expectations of TE intervention in nursing homes. 15 care providers will be enrolled to participate in face-to-face semi-structured interviews. These interviews will be transcribed verbatim and analyzed manually using thematic analysis. Secondly, results from the qualitative acceptability survey will be used to devise a questionnaire that will be conducted via computer-assisted telephone interviews involving 180 medical care providers. The findings from this questionnaire will provide a picture of the how this TE was globally perceived by medical providers. The questionnaire will collect socio-professional information and include specific questions evaluating intervention satisfaction (see additional files 1 and 2). Thirdly, a qualitative post-intervention survey will be conducted involving 20 medical care providers. The interviews will be transcribed and analyzed manually via thematic analysis. Lastly, quantitative and qualitative satisfaction survey results will be mixed and interpreted, with the latter aiming to form the basis of quantitative data findings [34].

#### Qualitative approach

Thematic analysis of the interviews will take place in two stages: 1) analysis of verbatim transcriptions to identify the themes that stand out in each one, followed by 2) transversal analysis of verbatim transcriptions to identify common themes and their frequency and single out concerns as per healthcare professional category.

#### Quantitative approach

Statistical analysis will essentially be of a descriptive nature and may include cross-tabulation of variables or even regression analysis to investigate factors associated with certain perception variables.

As of march 2020, the status of the trial is: recruiting. The study enrolment began in April 2019.

## Discussion

On account of iatrogenic disease that may stem from the utilization of prescribed medication and the resulting negative consequences for health in the elderly, better prescribing in this population has been recognized by the World Health Organization (WHO) in its recent report on aging and health as a high-priority area of focus [35]. Medication optimization in these patients can nevertheless prove to be challenging since there is a dearth of evidence-based medical data pertaining to the implementation of therapeutic guidelines in frail or even physically dependent elderly patients with multiple comorbidities and polypharmacy. A 2013 report by P. Verger on medication policy in nursing homes confirms that it is vital to promote regular medication review [36]. Medication optimization is a valuable means of checking whether each medication is still indicated for and of clinical benefit to the condition under consideration, of checking possible contraindications whether dosage is optimal, of questioning whether dosage form is appropriate (i.e. in the event of swallowing disorders) and whether a patient self-medicates. It also provides an overview of current medical conditions and highlights those requiring prompt treatment. Nursing home patients are an important target population for medication optimization in the elderly. Transport difficulties and time constraints on GP impede review of and improvements to drug prescribing. Indeed, healthcare professionals broadly observed that GP invest little time in medication management within nursing homes. With this in mind, the interest to offer a personalized medication review in nursing homes remain. In the literature, various studies have investigated the utility of structured medication review processes, but outcome measures are heterogeneous, and results vary widely. Medication review can detect medication-related problems in many patients, but evidence of clinical impact is scant. There is little evidence that medication reconciliation and medication review processes, as currently performed, significantly improve clinical outcomes [37]. The only significant change observed was a reduction in falls while no changes in mortality, resident behavior, hospital admission rates, functional status or cognitive skills have been noted so far [38–40]. We have therefore decided to evaluate the impact of TMR of nursing home patients on unplanned hospital admissions and other criteria, as assessed by a hospital team within the context of a randomized controlled study. This is a growing research field and fortunately similar studies are under way. The InTherAKT study is a single-arm study whose aim is to optimize communication between the healthcare professionals involved by specific training and by establishing a structured medication review process, and to improve medication appropriateness in line with the Medication Appropriateness Index for nursing home residents [41]. The HIOPP-3-iTBX study investigates the effects of pharmacist-led medication reviews linked with measures to strengthen interprofessional cooperation with regard to the quality of medication, health status and health care use of nursing home residents in a cluster randomized controlled trial [42]. The SCREAM study is a randomized study aimed at implementing a computerized clinical decision support system that analyzes nursing home resident medications in terms of interactions, appropriateness of dosage and other clinical data to minimize the time resources required for medication reviews by pharmacists and nursing home physicians [43]. On account of the mobility problems encountered by nursing home residents and the limited resources at the disposal of mobile geriatric care units to reach the various institutions, we have decided to intervene via telehealth program. Telemedicine is an alternative means of delivering care that calls for the same quality and safety standards as conventional healthcare. By analyzing all of the criteria involved in medication management (medication reconciliation, clinical and biological parameters, previous history etc.), telemedicine provides specific expert recommendations both remotely but also as closely as possible to where patients live. It is also instrumental in improving the efficiency and logistics of healthcare. Its goal is not to replace face-to-face medical treatment but to complement conventional healthcare. Rather than taking the place of customary medical practice, it constitutes a means of overcoming the challenges facing present-day healthcare provision. In our study, management decision-making lies firmly with the GP who is at liberty to accept or refuse the recommendations put forward. Thus, there is a risk of zero impact if GP refuse to at least partially take on board recommendations arising from TMR. Hence, we feel it is important to investigate how this intervention is perceived and received and to ascertain potential organizational and psychosocial factors prone to facilitate or on the contrary hamper its implementation, acceptability and impact on patients. The combination of both an acceptability survey and a satisfaction survey, the latter of which uses a mixed-method (qualitative and quantitative) approach, is particularly innovative. Indeed, in similar previous projects, feasibility/experience assessment was conducted using qualitative or quantitative methods alone [44, 45], or focused solely on post-intervention evaluation [46, 47]. Moreover, the WHO defines “acceptability to participants” as an essential ethical issue in study design [48]. Owing to the economic issues at stake in this new type of practice, we will also evaluate the impact in terms of cost-effectiveness of TMR as compared to standard healthcare. The benefits of collaboration between geriatric physicians and clinical pharmacists have already been borne out by other research teams [49, 50]. The feasibility of the proposed study relies on hospital teams that are already familiar with reconciliation and have received training in geriatric medication review. The number of nursing homes agreeing to participate means that only 20% of the residents will be required for inclusion, which appears perfectly feasible according to presentation of the present study to the coordinating physicians who will be in charge of inclusion. Moreover, additional nursing homes have expressed their desire to participate since the start of the study, making a total of 17 nursing homes. Financial compensation for each inclusion will be paid to coordinating physicians and, bearing in mind that TMR may mean that GP will need to take time to adjust their prescriptions, they too will receive payment equivalent to a home visit for each patient included in the experimental group. Data collection will be conducted by clinical research assistants to minimize any risks of missing data that may have occurred had we overburdened the coordinating physicians.

In the present study, we have attempted to keep the exclusion criteria for patients enrolled in the study to a minimum. Thus, patients under limited or general guardianship who are usually excluded from trials can perfectly well be included with the agreement of their legal representative. Likewise, we wished to include patients at both high and low risk of iatrogenic disease yet equally wished to be able to evaluate the impact of the intervention in terms of Trivalle’s score, which explains why randomization is stratified by the Trivalle’s risk score. On the other hand, for the purposes of investigating resident quality of life we decided to exclude patients with an MMSE score of less than 18. Changes to medication are equally more difficult to implement in patients suffering from severe cognitive impairment for fear of exacerbating their condition [51]. The long-term goal, should the benefits of medication review be proven, is to extend its use to all nursing home residents as part of telemedicine services.

By way of conclusion, the results from this randomized trial are expected to confirm that medication review via TE could be a worthwhile care management strategy for nursing home residents.

## Supplementary information

**Additional file 1.** GP interview guide.

**Additional file 2.** NH employee interview guide.

## Data Availability

Not applicable.

## Abbreviations

TE: Tele-Expertise
TMR: TeleMedication Review
MMSE: Mini Mental State Examination
GP: General Practitioner
PPP: Personalized Pharmaceutical Plan
QoL: Quality of Life
EQ-5D-3 L: EuroQol five-Dimension three-Level
EQ-VAS: EQ Visual Analog Scale
NPI: Neuro Psychiatric Inventory
ICER: Incremental Cost Effectiveness Ratios
QALYs: Quality-Adjusted Life Years
WHO: World Health Organization
