# Supplementary material for: Impact of medication review via tele-expertise on unplanned hospitalizations at 3 months of nursing homes patients (TEM-EHPAD): study protocol for a randomized controlled trial
Source: BMC Geriatr. 2020 Apr 20;20:147. doi: 10.1186/s12877-020-01546-3 (PMC7169005; doi:10.1186/s12877-020-01546-3)
Supplement: Supplementary file 1 — Additional file 1. GP interview guide. [file 12877_2020_1546_MOESM1_ESM.docx]

Additional file 1: GP interview guide (PHASE 1)

| - **Can you tell me about the drug prescriptions for nursing homes’ residents?**   Points to discuss:   - Specificities - Consultation frequency - End of hospitalization consultation - Help / external resources to prescribe to nursing homes’ residents - Relationship with pharmacists - Prescriptions re-evaluation frequency - Difficulties to re-evaluations? (poly-pathology, theoretical knowledge, pressure from patients, lack of time ...) - **Can you tell me about modifications of your patients’ drug prescriptions (for example, during hospitalization)?** - Type of modifications (Addition, withdrawal, dosage) - Have you been notified of these modifications? If yes, how (mail, email, phone call)? - Who made these modifications? Who warned you? - Feeling/views about those modifications - Understanding of those modifications - Maintaining of those modifications - **Overall, what do you think/how do you feel about the TEM-EPHAD project?** - Do you think it is appropriate to your needs? - Do you wish to have more information? - Expectations concerning :   **- Communication**  **- Prescription revision feedback (length, presentation)**   - **Do you have suggestions/advices to improve drug management of nursing homes’ residents?** |
| --- |
