# Supplementary material for: Impact of medication review via tele-expertise on unplanned hospitalizations at 3 months of nursing homes patients (TEM-EHPAD): study protocol for a randomized controlled trial
Source: BMC Geriatr. 2020 Apr 20;20:147. doi: 10.1186/s12877-020-01546-3 (PMC7169005; doi:10.1186/s12877-020-01546-3)
Supplement: Supplementary file 2 — Additional file 2. NH employee interview guide. [file 12877_2020_1546_MOESM2_ESM.docx]

Additional file 2 :NH employee interview guide (nurse, coordinating nurse, Phase 1)

| - **Can you tell me about the drug distribution for nursing home residents?**   Points to discuss:   - Specificities - Difficulties - Relationship with GP - Relationship with pharmacists - **Can you tell me about modifications of residents’ drug prescriptions (for example, during hospitalization)?** - Type of modifications (Addition, withdrawal, dosage) - Have you been notified of these modifications? If yes, how (mail, email, phone call)? - Who made these modifications? Who warned you? - Feeling/views about those modifications - Understanding of those modifications - Maintaining of those modifications - **Overall, what do you think/how do you feel about the TEM-EPHAD project?** - What are your expectations regarding this project? - Do you think it is appropriate to your needs? - Do you wish to have more information? - **Do you have suggestions/advices to improve drug management of nursing home’s residents?** |
| --- |
